# Prognostic nomogram based on the lymph node metastasis indicators for patients with bladder cancer: A SEER population‐based study and external validation

**DOI:** 10.1002/cam4.5475

**Published:** 2022-12-07

**Authors:** Shuai Li, Yicun Wang, Xiaopeng Hu

**Affiliations:** ^1^ Department of Urology Beijing Chao‐Yang Hospital, Capital Medical University Beijing China; ^2^ Institute of Urology Capital Medical University Beijing China

**Keywords:** bladder cancer, LODDS, lymph node metastasis, nomogram, prognosis, SEER

## Abstract

**Purpose:**

This study aimed to compare the prognostic value of multiple lymph node metastasis (LNM) indicators and to develop optimal prognostic nomograms for bladder cancer (BC) patients.

**Methods:**

BC patients were obtained from the Surveillance, Epidemiology, and End Results (SEER) database between 2004 and 2015, and randomly partitioned into training and internal validation cohorts. Genomic and clinical data were collected from The Cancer Genome Atlas (TCGA) as external validation cohort. The predictive efficiency of LNM indicators was compared by constructing multivariate Cox regression models. We constructed nomograms on basis of the optimal models selected for overall survival (OS) and cause‐specific survival (CSS). The performance of nomograms was evaluated with calibration plot, time‐dependent area under the curve (AUC) and decision curve analysis (DCA) in three cohorts. We subsequently estimated the difference of biological function and tumor immunity between two risk groups stratified by nomograms in TCGA cohort.

**Results:**

Totally, 10,093 and 107 BC patients were screened from the SEER and TCGA databases. N classification, positive lymph nodes (PLNs), lymph node ratio (LNR) and log odds of positive lymph nodes (LODDS) were all independent predictors for OS and CSS. The filtered models containing LODDS had minimal Akaike Information Criterion, maximal concordance indexes and AUCs. Age, LODDS, T and M classification were integrated into nomogram for OS, while nomogram for CSS included gender, tumor grade, LODDS, T and M classification. The nomograms were successfully validated in predictive accuracy and clinical utility in three cohorts. Additionally, the tumor microenvironment was different between two risk groups.

**Conclusions:**

LODDS demonstrated superior prognostic performance over N classification, PLN and LNR for OS and CSS of BC patients. The nomograms incorporating LODDS provided appropriate prediction of BC, which could contribute to the tumor assessment and clinical decision‐making.

## Graphical abstract

LODDS had better predictive accuracy than other lymph nodes metastasis indicators for BC patients. Nomograms including LODDS could improve the prediction of OS and CSS for BC patients.

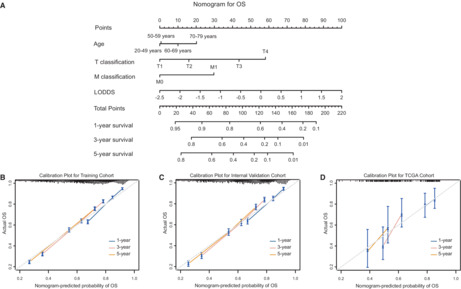

## 1 INTRODUCTION

Bladder cancer (BC) is the most common malignant tumor of urinary tract.^1^ Radical cystectomy with pelvic lymph node dissection (PLND) remains recommended treatment for patients with muscle‐invasive bladder cancer (MIBC) and high‐risk non‐muscle invasive bladder cancer.^2^, ^3^ The rate of lymph node metastasis (LNM) is up to 25% for MIBC patients, which is significantly associated with tumor recurrence and lower survival rates.^4^, ^5^ However, the assessment for LNM is still unsatisfactory which is most widely estimated on the basis of the American Joint Committee on Cancer (AJCC) tumor‐node‐metastasis (TNM) staging system.^6^ The AJCC N classification ignores the different number of dissected regional lymph nodes (LNs), which may decrease the accuracy of prognosis prediction.^7^ In addition, not only has the consensus of the optimal extent of PLND not been reached, there are individual differences in the pattern of regional LNM.^8^, ^9^ Therefore, novel LNM indicators are urgently needed for better treatment strategies.

In recent years, several LN prognostic factors were proposed to estimate the prognosis of BC patients, including the positive lymph nodes (PLNs) and the lymph node ratio (LNR).^10^, ^11^ Furthermore, the log odd of positive lymph nodes (LODDS) was proved to be more reliable for multiple tumor types.^12^, ^13^, ^14^ Jin et al. discovered that LODDS promoted better predictive efficiency for MIBC patients than AJCC N classification and LNR.^15^ However, this study did not explore the ability for predicting cause‐specific survival (CSS) or further establish available prognostic nomogram for clinical usage. To our knowledge, there is no report on the evaluation of novel LNM indicators for predicting both overall survival (OS) and CSS of BC patients.

The present study aimed to compare the prognostic values among different LN status factors of BC patients by analyzing data from the Surveillance, Epidemiology, and End Results (SEER) database. Subsequently, we established novel nomograms incorporating LODDS for predicting OS and CSS, and successfully validated them in internal and external validation cohorts.

## 2 METHODS

### 2.1 Data source

We collected patients from the SEER database (SEER*Stat version 8.3.9.2) of the National Cancer Institute (NCI) program, which is one of the most representative tumor databases and covers approximately 28% of the US population.^16^, ^17^ We obtained permission to access the database using the reference number 15912‐Nov2020. In addition, we collected the genomic and clinical information of BC from The Cancer Genome Atlas (TCGA, https://tcga‐data.nci.nih.gov/tcga/). Ethics approval for this study was exempt as all data were download from publicly databases.

### 2.2 Study population

**FIGURE 1:**
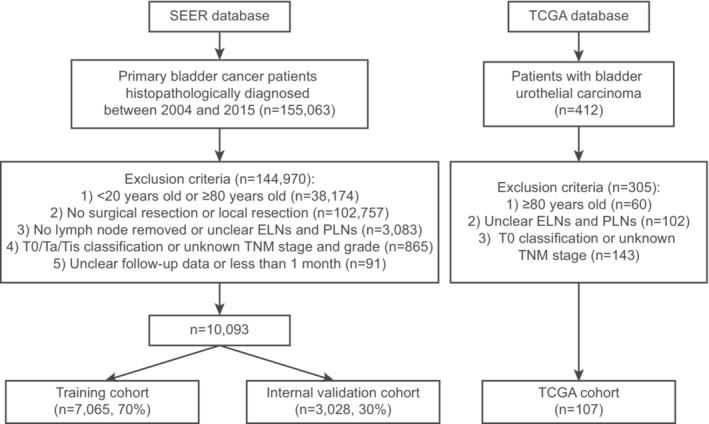
Flowchart illustrating patient selection of this study. ELN, examined lymph node; PLN, positive lymph node; SEER, Surveillance, Epidemiology, and End Results; TCGA, The Cancer Genome Atlas.

Patients diagnosed with primary bladder cancer (ICD‐O‐3/WHO 2008: “Urinary Bladder”) between 2004 and 2015 were enrolled into the study. The exclusion criteria for data extraction were (1) patients aged <20 years or ≥ 80 years at diagnosis; (2) patients with no surgery or undergoing local resection; (3) patients with no LN removed or with unclear examined LNs (ELNs) and PLNs; (4) patients with T0/Ta/Tis classification or with unclear AJCC TNM stage and tumor grade; (5) patients with unclear survival data or survival time less than 1 month. Finally, we included 10,093 BC patients after cystectomy or radical cystectomy from SEER and partitioned them into training cohort (*n* = 7065, 70%) and internal validation cohort (*n* = 3028, 30%), using a random sampling method. Additionally, we obtained 107 BC samples from TCGA based on the exclusion criteria as an external validation cohort. Figure 1 displays the selection process of patients.

### 2.3 Measurement of variables

We collected variables of patients including age at diagnosis, gender, T/N/M classification, tumor grade, the amount of regional ELNs and PLNs, survival time and status. The tumor stage was based on the sixth edition of AJCC staging system, which was adapted to SEER‐derived patients diagnosed from 2004 to 2015. Low‐ and high‐grade tumors in TCGA were considered as grade I‐II and III‐IV in SEER database, respectively. The LNR was defined as the ratio of the amount of PLNs and ELNs. The LODDS was formulated by log [(PLNs +0.5) / (ELNs – PLNs +0.5)]. To avoid division by zero error, we added 0.5 to both numerator and denominator.

The primary and secondary endpoints were OS and CSS, which were shown as “COD to site recode” and “SEER cause‐specific death classification” in SEER database, respectively. Of note, the data from the SEER and TCGA were downloaded on December 9, 2021.

### 2.4 Independence of LN status indicators

We selected the clinicopathological predictors with univariable Cox regression analyses through “survival” R package for OS and CSS in training cohort. To further assess the predictive values, each LN status factor (including N classification, PLN, LNR and LODDS) was integrated into multivariate regression models together with other risk variables with *p* < 0.05 in univariate analyses, respectively.

### 2.5 Comparison of predictive performance among LN status indicators

Backward stepwise selection (via “MASS” R package) was utilized into the above models using Akaike Information Criterion (AIC) as the stopping rule, respectively.^18^ The model with minimum AIC was selected as the optimal one. The predictive efficiency of these filtered models incorporating different LN factors was compared using AIC, bootstrapped concordance index (C‐index) and area under the curve (AUC) via “riskRegression” R package.

### 2.6 Construction and validation of nomograms

Variables included in the filtered models with the highest accuracy were integrated to develop nomograms for predicting OS and CSS in training cohort (via “rms” R package), respectively. The efficiency of nomograms was evaluated by bootstrapped C‐indexes, time‐dependent AUCs and calibration plots in training, internal validation and TCGA cohorts. Additionally, we preformed decision curve analysis (DCA) to assess the net benefit and clinical performance of the nomograms using the “ggDCA” R package.^19^

### 2.7 Survival risk classifiers established by nomograms

The multivariate Cox regression formulas of the nomograms for OS and CSS formed in training cohort were applied into patients in three cohorts using “nomogramFormula” R package. All patients were divided into high‐ and low‐risk groups according to the total points calculated via “survminer” R package. The Kaplan–Meier (K‐M) method was used to assess the survival difference of OS and CSS between two risk groups.

### 2.8 Functional enrichment

Differential expression genes (DEGs) from TCGA were searched for by comparing high‐ and low‐risk groups with the threshold of |log fold change| >1 and *p* < 0.05 (via “limma” R package). We performed enrichment analyses for Gene ontology (GO) and Kyoto Encyclopedia of Genes and Genomes (KEGG) with Metascape (https://metascape.org/gp/index.html) to further verify the biological function enrichment.^20^ The number of min overlaps, min enrichment and P value cutoff were set at 3, 1.5 and 0.01, respectively. Gene Set Enrichment Analysis (GSEA) software (Version 4.2.0) was also used to explore the function difference between two risk groups.^21^ Hallmark gene sets were analyzed and the number of permutations was set at 1000. Normalized Enrichment Score (NES) > 1.5 and *p* < 0.05 were considered statistically significant.

### 2.9 Estimation of tumor immune infiltration

To estimate the tumor microenvironment (TME), the stromal and immune scores of each sample in TCGA cohort were analyzed through the “estimate” R package.^22^ We calculated the relative proportion of 22 human immune cell subsets by the CIBERSORT algorithm through “preprocessCore” R package.^23^ Additionally, we also investigated the relationship between the risk classifiers and the genes related to immune checkpoint inhibitors (ICIs) via violin plot visualization.

### 2.10 Statistical analysis

Continuous variables with non‐normal distribution were reported as median with interquartile range (IQR) and categorical variables were presented as frequencies with percentages. Statistical significance was achieved with a two‐sided *p* value less than 0.05. All statistical analyses were performed using R software (version 4.0.5).

## 3 RESULTS

### 3.1 Patient characteristics and survival

The patients' characteristics of training, internal validation and TCGA cohorts are shown in Table 1. The median follow‐up time for entire SEER and TCGA databases were 100 (95% confidence interval [CI]: 99–102) months and 37 (95% CI: 30–54) months. Patients in the SEER and TCGA databases had the 5‐year OS rates of 51% (95% CI: 50%–52%) and 45% (95% CI: 34%–59%), respectively. The detailed distributions of the LN indicators are shown in Figure S1.

**TABLE 1:** Clinical and pathologic characteristics of patients with BC in three cohorts

Characteristics	Training (*n* = 7065)	Internal validation (*n* = 3028)	TCGA (*n* = 107)
Age (year), *n* (%)			
20–49	492 (7.0)	233 (7.7)	2 (1.9)
50–59	1527 (21.6)	684 (22.6)	18 (16.8)
60–69	2701 (38.2)	1107 (36.6)	43 (40.2)
70–79	2345 (33.2)	1004 (33.2)	44 (41.1)
Gender, *n* (%)			
Female	1723 (24.4)	741 (24.5)	23 (21.5)
Male	5342 (75.6)	2287 (75.5)	84 (78.5)
T classification, *n* (%)			
T1	687 (9.7)	292 (9.6)	0 (0)
T2	2789 (39.5)	1183 (39.1)	26 (24.3)
T3	2436 (34.5)	1037 (34.2)	61 (57.0)
T4	1153 (16.3)	516 (17.0)	20 (18.7)
N classification, *n* (%)			
N0	5031 (71.2)	2187 (72.2)	70 (65.4)
N1	1001 (14.2)	424 (14.0)	15 (14.0)
N2	993 (14.1)	406 (13.4)	20 (18.7)
N3	40 (0.6)	11 (0.4)	2 (1.9)
M classification, *n* (%)			
M0	6692 (94.7)	2903 (95.9)	103 (96.3)
M1	373 (5.3)	125 (4.1)	4 (3.7)
Grade, *n* (%)			
Grade I/II	440 (6.2)	178 (5.9)	NA
Grade III/IV	6625 (93.8)	2850 (94.1)	NA
Low	NA	NA	1 (0.9)
High	NA	NA	106 (99.1)
PLN, median (IQR)	0 (0, 1.00)	0 (0, 1.00)	0 (0, 1.00)
LNR, median (IQR)	0 (0, 0.05)	0 (0, 0.04)	0 (0, 0.05)
LODDS, median (IQR)	−1.28 (−1.59, −0.85)	−1.29 (−1.59, −0.85)	−1.28 (−1.57, −0.95)
Overall survival, *n* (%)			
Alive	3042 (43.1)	1311 (43.3)	60 (56.1)
Dead	4023 (56.9)	1717 (56.7)	47 (43.9)
Cause‐specific survival, n (%)			
Alive or dead of other cause	4087 (57.8)	1768 (58.4)	75 (70.1)
Dead of BC	2978 (42.2)	1260 (41.6)	32 (29.9)
Follow‐up time (month), Median (95% CI)	100 (99, 103)	100 (97, 104)	37 (30, 54)

Abbreviations: BC, bladder cancer; CI, confidence interval; IQR, interquartile range; LNR, lymph node ratio; LODDS, log odds of positive lymph node; PLN, positive lymph node.

### 3.2 Prognostic analyses for OS and CSS

The detailed results of the univariate Cox regression analyses in training cohort are demonstrated in Table 2. Age, gender, T, N, M classification, grade, PLN (hazard ratio [HR]: 1.07, 95% CI: 1.06–1.07), LNR (HR: 6.61, 95% CI: 5.90–7.42) and LODDS (HR: 2.12, 95% CI: 2.03–2.22) were significant risk factors for OS. Gender, T, N, M classification, grade, PLN (HR: 1.07, 95% CI: 1.07–1.08), LNR (HR: 8.27, 95% CI: 7.31–9.35) and LODDS (HR: 2.36, 95% CI: 2.25–2.48) were significant prognostic factors for CSS.

**TABLE 2:** Univariate Cox regression analyses for predicting OS and CSS in training cohort

Characteristics	OS		CSS	
	HR (95% CI)	*p* value	HR (95% CI)	*p* value
Age (year)				
20–49	Reference		Reference	
50–59	1.00 (0.87–1.16)	0.993	0.87 (0.74–1.01)	0.065
60–69	1.21 (1.06–1.38)	0.006*	0.97 (0.84–1.12)	0.656
70–79	1.52 (1.33–1.74)	<0.001*	1.09 (0.94–1.26)	0.238
Gender (Male/Female)	0.92 (0.86–0.99)	0.019*	0.83 (0.76–0.90)	<0.001*
T classification				
T1	Reference		Reference	
T2	1.46 (1.27–1.68)	<0.001*	1.73 (1.42–2.09)	<0.001*
T3	3.10 (2.70–3.56)	<0.001*	4.42 (3.67–5.33)	<0.001*
T4	4.90 (4.24–5.67)	<0.001*	7.42 (6.12–9.00)	<0.001*
N classification				
N0	Reference		Reference	
N1	2.18 (2.01–2.37)	<0.001*	2.68 (2.44–2.94)	<0.001*
N2	3.04 (2.81–3.30)	<0.001*	3.83 (3.50–4.19)	<0.001*
N3	5.61 (4.03–7.80)	<0.001*	7.12 (5.06–10.01)	<0.001*
M classification (M1/M0)	3.66 (3.27–4.10)	<0.001*	4.31 (3.82–4.85)	<0.001*
Grade (Grade III‐IV/I‐II)	1.15 (1.01–1.31)	0.037*	1.25 (1.07–1.47)	0.006*
PLN	1.07 (1.06–1.07)	<0.001*	1.07 (1.07–1.08)	<0.001*
LNR	6.61 (5.90–7.42)	<0.001*	8.27 (7.31–9.35)	<0.001*
LODDS	2.12 (2.03–2.22)	<0.001*	2.36 (2.25–2.48)	<0.001*

Abbreviations: CI, confidence interval; CSS, cause‐specific survival; HR, hazard ratio; LNR, lymph node ratio; LODDS, log odds of positive lymph node; OS, overall survival; PLN, positive lymph node.

* Represented *p* value < 0.05.

We further performed multivariate analyses and generated prognostic models including different LN indicators, respectively. Briefly, N classification, PLN, LNR and LODDS were all independent risk factors for OS (Table 3) and CSS (Table 4). Besides, age, T and M classification were independent prognostic factors for OS; gender, T and M classification were independent risk factors for CSS.

**TABLE 3:** Multivariate Cox regression analyses for predicting OS in training cohort

Characteristic	N classification		PLN		LNR		LODDS	
	HR (95% CI)	*p* value	HR (95% CI)	*p* value	HR (95% CI)	*p* value	HR (95% CI)	*p* value
Age (year)								
20–49	Reference		Reference		Reference		Reference	
50–59	1.01 (0.88–1.17)	0.868	1.00 (0.87–1.16)	0.990	1.00 (0.87–1.16)	0.960	1.02 (0.88–1.17)	0.821
60–69	1.27 (1.11–1.45)	0.001*	1.24 (1.08–1.42)	0.002*	1.24 (1.09–1.42)	0.002*	1.25 (1.09–1.43)	0.001*
70–79	1.67 (1.46–1.92)	<0.001*	1.59 (1.39–1.82)	<0.001*	1.58 (1.38–1.81)	<0.001*	1.57 (1.37–1.80)	<0.001*
Gender (Male/Female)	0.98 (0.91–1.05)	0.586	0.97 (0.90–1.04)	0.357	0.97 (0.90–1.04)	0.418	0.97 (0.90–1.04)	0.435
T classification								
T1	Reference		Reference		Reference		Reference	
T2	1.40 (1.21–1.61)	<0.001*	1.46 (1.26–1.68)	<0.001*	1.44 (1.25–1.66)	<0.001*	1.43 (1.24–1.64)	<0.001*
T3	2.50 (2.17–2.87)	<0.001*	2.91 (2.53–3.34)	<0.001*	2.75 (2.39–3.16)	<0.001*	2.64 (2.29–3.03)	<0.001*
T4	3.53 (3.04–4.11)	<0.001*	4.31 (3.72–5.00)	<0.001*	3.84 (3.31–4.46)	<0.001*	3.65 (3.15–4.24)	<0.001*
M classification (M1/M0)	2.14 (1.90–2.40)	<0.001*	2.20 (1.94–2.49)	<0.001*	1.99 (1.77–2.25)	<0.001*	1.94 (1.72–2.19)	<0.001*
Grade (Grade III‐IV/I‐II)	0.94 (0.83–1.08)	0.401	1.03 (0.90–1.17)	0.694	1.01 (0.88–1.15)	0.919	1.00 (0.87–1.14)	0.977
N classification								
N0	Reference							
N1	1.68 (1.54–1.83)	<0.001*						
N2	2.07 (1.89–2.25)	<0.001*						
N3	3.22 (2.30–4.49)	<0.001*						
PLN			1.03 (1.02–1.04)	<0.001*				
LNR					3.29 (2.88–3.75)	<0.001*		
LODDS							1.64 (1.56–1.72)	<0.001*

Abbreviations: CI, confidence interval; HR, hazard ratio; LNR, lymph node ratio; LODDS, log odds of positive lymph node; OS, overall survival; PLN, positive lymph node.

* Represented *p* value < 0.05.

**TABLE 4:** Multivariate Cox regression analyses for predicting CSS in training cohort

Characteristic	N classification		PLN		LNR		LODDS	
	HR (95% CI)	*p* value	HR (95% CI)	*p* value	HR (95% CI)	*p* value	HR (95% CI)	*p* value
Gender (Male/Female)	0.88 (0.81–0.96)	0.003*	0.86 (0.8–0.94)	<0.001*	0.87 (0.81–0.95)	0.001*	0.88 (0.81–0.95)	0.002*
T classification								
T1	Reference		Reference		Reference		Reference	
T2	1.60 (1.32–1.94)	<0.001*	1.70 (1.40–2.06)	<0.001*	1.67 (1.38–2.03)	<0.001*	1.66 (1.37–2.01)	<0.001*
T3	3.30 (2.73–3.99)	<0.001*	4.04 (3.35–4.87)	<0.001*	3.78 (3.13–4.56)	<0.001*	3.60 (2.98–4.35)	<0.001*
T4	4.76 (3.91–5.80)	<0.001*	6.17 (5.08–7.50)	<0.001*	5.38 (4.42–6.55)	<0.001*	5.08 (4.17–6.18)	<0.001*
M classification (M1/M0)	2.26 (2.00–2.56)	<0.001*	2.35 (2.06–2.69)	<0.001*	2.11 (1.85–2.39)	<0.001*	2.03 (1.79–2.30)	<0.001*
Grade (Grade III‐IV/I‐II)	1.06 (0.90–1.25)	0.472	1.18 (1.00–1.38)	0.048*	1.15 (0.98–1.35)	0.094	1.13 (0.96–1.33)	0.138
N classification								
N0	Reference							
N1	1.87 (1.70–2.06)	<0.001*						
N2	2.35 (2.13–2.59)	<0.001*						
N3	3.62 (2.56–5.10)	<0.001*						
PLN			1.03 (1.03–1.04)	<0.001*				
LNR					3.74 (3.24–4.31)	<0.001*		
LODDS							1.75 (1.66–1.85)	<0.001*

Abbreviations: CI, confidence interval; CSS, cause‐specific survival; HR, hazard ratio; LNR, lymph node ratio; LODDS, log odds of positive lymph node; PLN, positive lymph node.

* Represented *p* value < 0.05.

### 3.3 Comparison of N classification, PLN, LNR and LODDS

The comparison of LN status indicators in training cohort is shown in Table 5. After backward stepwise selection for the above prognostic models, the AICs of the filtered models containing LODDS for OS (65458.57) and CSS (48732.61) were lowest than that of other LN factors (Table S1). The C‐indexes of the filtered models containing LODDS were higher over that of N classification, PLN and LNR for OS (0.705) and CSS (0.727). Meanwhile, the 1‐, 3‐ and 5‐year AUCs of the selected model including LODDS were higher than that of others except for the 5‐year AUC of OS (LODDS: 0.759 [0.747–0.770] vs. N classification: 0.762 [0.751–0.774]). Taken together, the results indicated that the selected models containing LODDS had more enhanced predictivity for OS and CSS, and LODDS might act as the strongest predictive indicator over N classification, PLN and LNR.

**TABLE 5:** Prognostic efficiency of different lymph node status indicators in training cohort

Endpoint	Filtered model	C‐index	AIC	AUC		
				1‐year	3‐year	5‐year
Overall survival	N classification	0.702	65511.93	0.761 (0.747–0.775)	0.767 (0.756–0.779)	0.762 (0.751–0.774)
	PLN	0.690	65764.07	0.756 (0.742–0.770)	0.747 (0.735–0.759)	0.741 (0.729–0.754)
	LNR	0.699	65567.82	0.766 (0.752–0.780)	0.760 (0.749–0.771)	0.753 (0.741–0.764)
	LODDS	0.705	65458.57	0.770 (0.756–0.784)	0.768 (0.756–0.779)	0.759 (0.747–0.770)
Cause‐specific survival	N classification	0.721	48755.03	0.771 (0.756–0.786)	0.779 (0.767–0.791)	0.775 (0.763–0.787)
	PLN	0.712	49048.33	0.768 (0.753–0.783)	0.765 (0.753–0.777)	0.759 (0.747–0.772)
	LNR	0.721	48841.40	0.778 (0.763–0.793)	0.777 (0.765–0.789)	0.770 (0.758–0.782)
	LODDS	0.727	48732.61	0.783 (0.769–0.798)	0.783 (0.771–0.794)	0.775 (0.763–0.788)

Abbreviations: AIC, Akaike information criterion; AUC, area under the curve; C‐index, concordance index; LNR, lymph node ratio; LODDS, log odds of positive lymph node; PLN, positive lymph node.

### 3.4 Construction and validation of nomograms

**FIGURE 2:**
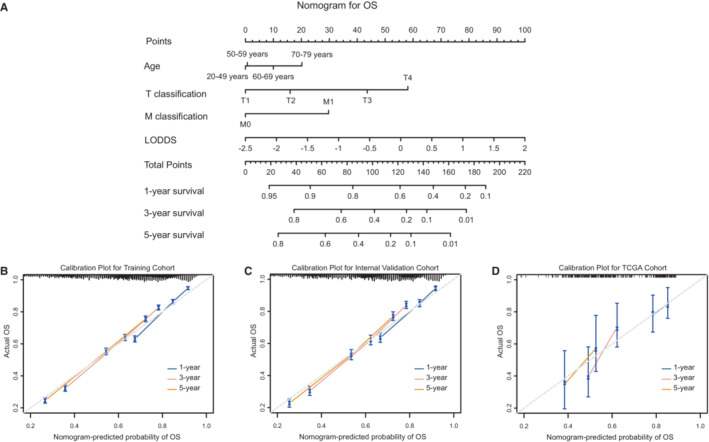
Nomogram for OS of BC patients. (A) Prediction for 1‐, 3‐ and 5‐year OS of nomogram. Calibration plots for 1‐, 3‐ and 5‐year in training (B), internal validation (C) and TCGA cohorts (D). BC, bladder cancer; LODDS, log odds of positive lymph node;OS, overall survival; TCGA, The Cancer Genome Atlas.

**FIGURE 3:**
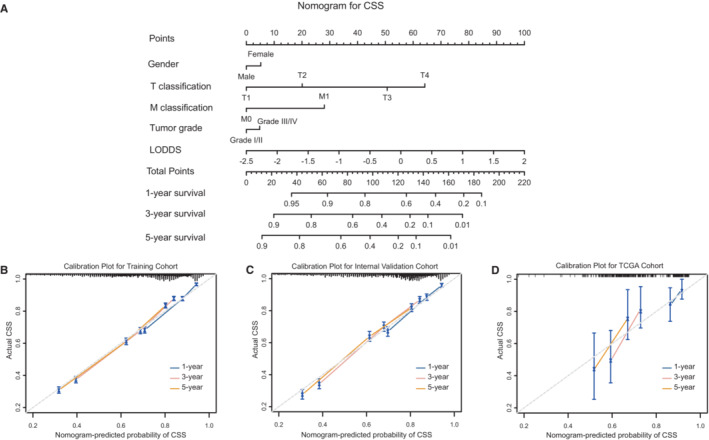
Nomogram for CSS of BC patients. (A) Prediction for 1‐, 3‐ and 5‐year CSS of nomogram. Calibration plots for 1‐, 3‐ and 5‐year in training (B), internal validation (C) and TCGA cohorts (D). BC, bladder cancer; CSS: cause‐specific survival; LODDS, log odds of positive lymph node; TCGA, The Cancer Genome Atlas.

**FIGURE 4:**
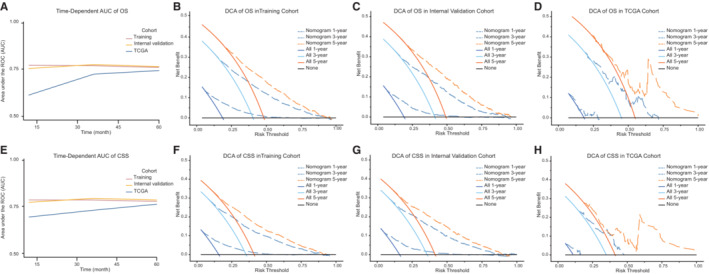
Evaluation of the nomograms for BC patients with AUC and DCA. The time‐dependent AUC for OS (A) and CSS (E) in three cohorts. Decision curves for predicting 1‐, 3‐ and 5‐year OS (B‐D) and CSS (F‐H) in training, internal validation and TCGA cohorts. Solid black line: assume no patients need clinical intervention, net benefit is zero. Solid color lines: assume all patients need receive clinical intervention. Dotted color lines: net benefits of the nomogram for predicting 1‐, 3‐ and 5‐year survival when patients receive intervention if predictions exceed the threshold. AUC, area under the curve; BC, bladder cancer; CSS, cause‐specific survival; DCA, decision curve analysis; OS, overall survival; ROC, receiver operating characteristic; TCGA, The Cancer Genome Atlas.

We developed nomograms based on the selected models containing LODDS in training cohort. As results, age, LODDS, T and M classification were incorporated into final nomogram for predicting OS (Figure 2A); gender, tumor grade, LODDS, T and M classification were included in nomogram for CSS (Figure 3A). Calibration plots of the three cohorts are displayed in Figure 2B‐D and Figure 3B‐D, indicating excellent agreement between predictive and actual observation for OS and CSS. The time‐dependent AUC values of the nomograms for OS (Figure 4A) and CSS (Figure 4E) demonstrated more stable accuracy and better predicting efficiency over time. Besides, the DCA curves of nomograms in three cohorts for OS (Figure 4B‐D) and CSS (Figure 4F‐H) are displayed. *X*‐axis represented threshold probabilities, and net benefit was plotted on the *y*‐axis. The net benefit achieved by making decision on the basis of predictive models balanced the risk of real mortality with the unnecessary intervention of false prediction. The DCA curves indicated that the nomogram added more net benefits compared both the treat‐all‐patients scheme and treat‐none scheme. We simultaneously evaluated detailed C‐indexes and 1‐, 3‐ and 5‐year AUCs values of nomograms in each cohort (Table S2). The above results revealed both appreciable reliability and clinical practicality of the prognostic nomograms.

### 3.5 Survival risk classifiers based on nomograms

**FIGURE 5:**
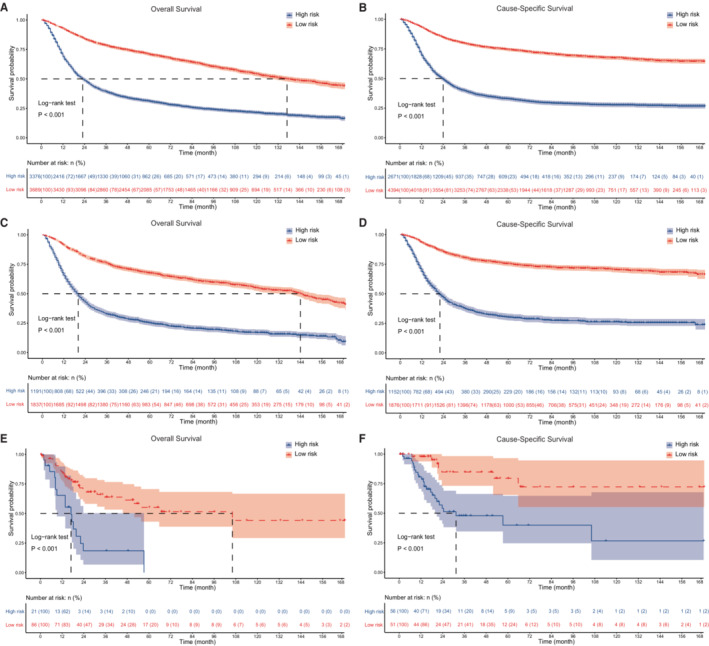
Kaplan–Meier (K‐M) analyses for BC patients classified by nomograms. (A, B) K‐M curves for OS and CSS in training cohort. (C, D) K‐M curves for OS and CSS in internal validation cohort. (E, F) K‐M curves for OS and CSS in TCGA cohort. BC, bladder cancer; CSS, cause‐specific survival; OS, overall survival; TCGA, The Cancer Genome Atlas.

To further verify the performance of nomograms, we divided patients into high‐ and low‐risk groups based on the total points calculated by nomograms for OS and CSS. The cutoff values of the total points were 100.57 for OS and 82.94 for CSS, respectively (Figure S2). K‐M curves showed significant distinction in survival outcomes stratified by the risk classifiers for OS and CSS in three cohorts (Figure 5).

### 3.6 Pathway enrichment analyses

**FIGURE 6:**
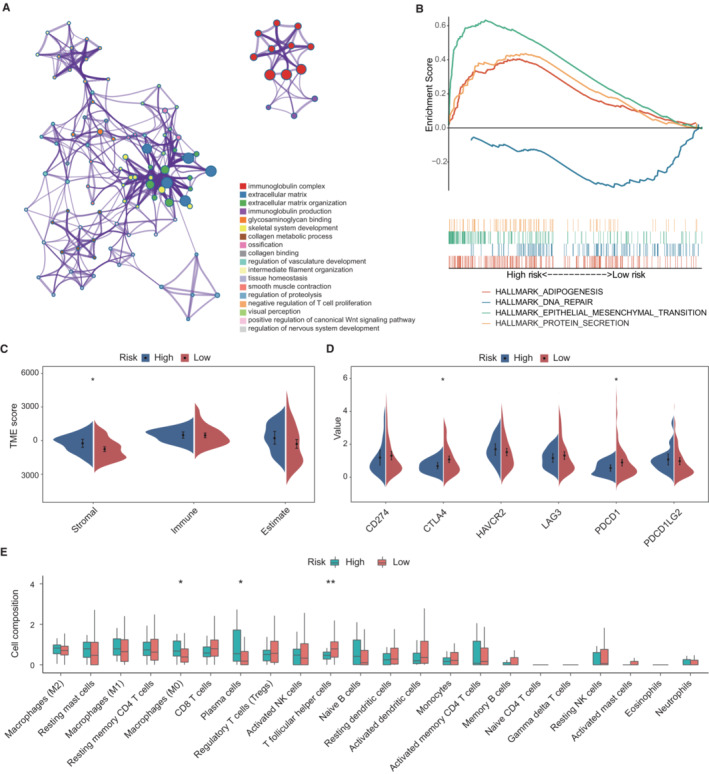
Analyses of pathway enrichment and tumor immunity of the risk classifier stratified by OS. (A) Network plot of the enriched terms clustered by Metascape. (B) The significantly enriched Hallmark gene sets performed by Gene Set Enrichment Analysis. (C) The stromal and immune scores between two groups. (D) Different expression of ICIs‐related genes among risk groups. (E) The different proportion of tumor‐infiltrating cells between two groups. * represented *p* value <0.05; ** represented *p* value < 0.01; ICIs, immune checkpoint inhibitors; OS, overall survival; TME, tumor microenvironment.

After standardization among the RNA‐seq in TCGA, we extracted 78 DEGs based on the risk classifier for OS. The representative statistically enriched pathways (both GO and KEGG) were clustered together and shown with a network plot (Figure 6A). The results showed that the DEGs were associated with immunoglobulin complex, extracellular matrix (ECM) organization, immunoglobulin production, and so on. In additional, GSEA analyses was performed using Hallmark database, illustrating that high‐risk group was positively associated with epithelial mesenchymal transition, adipogenesis and protein secretion (Figure 6B). In contrast, the DNA reparation pathway was enriched in low‐risk group. Meanwhile, we also selected 84 DEGs based on the risk classification for CSS, and the results of enrichment analyses are shown in Figure S3A,B.

### 3.7 The relationship between risk classifiers and tumor immunity

We investigated the TME and calculated stromal and immune scores for each BC sample based on the risk classifier for OS. The higher stromal score was observed in samples of high‐risk group (Figure 6C). We further investigated the differential relative proportion of immune infiltrating cells between two groups. The results showed the high‐risk group was more abundant in M0 macrophages and plasma cells, whereas T follicular helper cells had higher infiltration in low‐risk patients (Figure 6E). The correlation between risk classifier and ICI‐related genes were subsequently analyzed, and the expression of *CTLA4* and *PDCD1* were higher in low‐risk group (Figure 6D). The relationship between immune infiltration and the risk classifier based on CSS are presented in Figure S3C‐E.

## 4 DISCUSSION

Despite improvement in diagnosis and surgery, bladder cancer remains a heterogeneous malignancy with poor prognosis.^6^, ^24^ Individual patients within same AJCC stage vary widely in survival outcomes. Additionally, LNM is of critical importance to the prognosis of BC, while the N classification based on AJCC staging system is insufficient to accurately evaluate LNM.^4^, ^25^ Therefore, there is an urgently need to find novel LN status indicators to assess LNs involvement and stratify BC patients for individualized management.

Considering this situation, several modified LN status factors has been proposed to predict the survival of BC, including PLN, LNR and LODDS.^10^, ^11^, ^15^ Our study demonstrated that all four LN indicators were independent prognostic factors for OS and CSS in BC. We further compared the predictive values among N classification, PLN, LNR and LODDS. As results, selected models containing LODDS with minimal AICs, maximal C‐indexes and AUCs were considered as optimal prognostic models for OS and CSS, indicating that LODDS had better discrimination capability in predicting prognosis of BC patients.

The N classification of AJCC staging system evaluates LN status mainly by detecting in certain regional areas, which was unsatisfactory due to the unclear number of negative and removed LNs.^26^, ^27^ The number of PLNs was an unfavorable risk factor for cancer patients.^28^ Although more LN dissection could lead to a smaller probability of undiscovered PLNs, the threshold for LN dissection or the extent of PLND varied considerably and had not achieved consensus at present.^8^, ^29^, ^30^ LNR has been shown to reflect the progression of disease and to be a prognostic factor for BC.^4^, ^8^, ^31^ However, the association between LNR and tumor prognosis remains controversial when all or none examined LNs exhibited metastasis, especially with insufficient regional LNs removed.^32^ Compared with the above, LODDS could reflect both the absolute number of PLNs and negative LNs, indicating that it could stratify patients categorized as N0 classification (no regional LNM) and patients with all examined LNs metastasized.^29^, ^33^, ^34^

The current study suggested that the prognostic models with LODDS had best predictive capability for OS and CSS, which was similar to Jin et al.'s study for OS of BC patients. However, we paid more attention to clinical utilization and constructed two novel nomograms incorporating LODDS for predicting OS and CSS of BC patients for the first time. Then, the nomograms were further validated in internal and external validation cohorts. The calibration curves showed stable linearity and appropriate efficacy of the nomograms, and the calculated C‐indexes and time‐dependent AUCs were most above 0.70 and 0.75 in three cohorts, respectively. In terms of clinical utility, the DCA curves demonstrated that the nomogram showed consistent larger net benefit across a broad range of threshold probabilities. We believed that the nomograms had satisfactory applicability in predicting survival for BC patients. Taken together, better predictive accuracy and clinical validity were verified in our nomograms compared with AJCC and other LN status systems.

Furthermore, risk classifiers for OS and CSS were established on basis of the total scores from nomograms, stratifying BC patients into different risk groups. Patients in two high‐risk groups had worse survival in each cohort. We subsequently explored the biological function between the two groups. The results showed that there were major differences in immunoglobulin production and secretion, ECM organization, epithelial mesenchymal transition, adipogenesis and DNA reparation. It has been reported that the immunoglobulin, produced by B lymphocytes and plasma cells in response to immunogen, was associated with the prognosis in solid tumors.^35^, ^36^ ECM is a fundamental node of cell‐extrinsic metabolic regulation and the organization of ECM was related to tumorigenesis and migration.^37^, ^38^ The other enrichment pathways were also involved in the progression of multiple tumors.^39^, ^40^, ^41^ In addition, the TME and the sensitivity to ICI were evaluated in two groups. Noticeably, high‐risk patients had higher stroma scores, consistent with previous studies in BC patients.^42^, ^43^ The stromal cell alteration was previously shown to affect the development of organ‐specific metastasis.^44^ The extracellular vesicles derived from tumor cells could be transferred to adjacent organs and internalized by stromal cells, developing a metastatic‐designated microenvironment. Furthermore, specific stromal cells like cancer‐associated fibroblasts contribute to the upregulation of multiple cytokines and the remodeling of ECM, which could facilitate the tumor invasion and metastasis.^45^ The analysis of immune cell infiltration suggested that the high‐risk group had a higher infiltration of M0 macrophages and plasma cells, while T follicular helper cells were more abundant in low‐risk group. The high proportion of M0 macrophages, indicating a higher level of inflammatory activation, was correlated with poor prognosis of BC patients.^46^, ^47^ It has previously been reported that more recruitment of naïve M0 macrophages is associated with early LNM, which could prevent T cells from attacking tumor cells and secrete growth factors promoting tumor angiogenesis.^48^, ^49^ T follicular helper cells might contribute to the maintenance of a protective immune response, which was proved to be associated with better prognosis in BC.^50^, ^51^ Furthermore, we observed higher expression levels of *CTLA4* and *PDCD1* in low‐risk group, suggesting BC patients with low risk points based on OS classifier might benefit more from immunotherapy.^52^ Collectively, these results explained the possible mechanisms from the perspective of tumor biological and immune function and further corroborated the validity of our risk classifiers for OS and CSS.

Indeed, as a retrospective study, we need prospective and multicenter study to verify the prognostic value of LODDS. Secondly, the details of surgical approach, such as the extent of LN dissection at specific nodal level are not recorded in detail, which is worth further investigation. Despite these limitations, our study proves better predictive value of LODDS and firstly incorporates it into prognostic nomograms for both OS and CSS in BC patients.

## 5 CONCLUSIONS

We confirmed that LODDS had better predictive accuracy compared with other LNM indicators for BC patients after surgery. Novel nomograms containing LODDS for predicting OS and CSS were established based on SEER database and successfully validated in external dataset, which could assist urologists with more accurate therapeutic decision and personalized follow‐up management for BC patients.

## AUTHOR CONTRIBUTIONS

**Shuai Li:** Conceptualization (lead); data curation (lead); formal analysis (lead); investigation (lead); methodology (lead); project administration (lead); visualization (lead); writing – original draft (lead); writing – review and editing (lead). **Yicun Wang:** Conceptualization (supporting); data curation (supporting); formal analysis (supporting); investigation (supporting); methodology (supporting); project administration (supporting); validation (supporting); writing – review and editing (supporting). **Xiaopeng Hu:** Conceptualization (supporting); data curation (supporting); formal analysis (supporting); investigation (supporting); methodology (supporting); project administration (supporting); supervision (supporting); validation (supporting); writing – review and editing (supporting).

## CONFLICT OF INTEREST

The authors declared no conflicts of interest.

## ETHICAL APPROVAL STATEMENT

Ethical approval was not needed in this retrospective study, as all data abstracted from the public‐used databases was anonymous.

## Supporting information


Figure S1.

Figure S2.

Figure S3.

Table S1.

Table S2.

## Data Availability

This article is based on available data from the SEER (https://seer.cancer.gov/) and TCGA (https://tcga‐data.nci.nih.gov/tcga/) databases. We have got permission to access the SEER database from National Cancer Institute (ID 15912‐Nov2020).
