# Supplementary material for: Prognostic nomogram based on the lymph node metastasis indicators for patients with bladder cancer: A SEER population‐based study and external validation
Source: Cancer Med. 2022 Dec 7;12(6):6853–66. doi: 10.1002/cam4.5475 (PMC10067030; doi:10.1002/cam4.5475)
Supplement: Supplementary file 1 — Figure S1. [file CAM4-12-6853-s002.pdf]

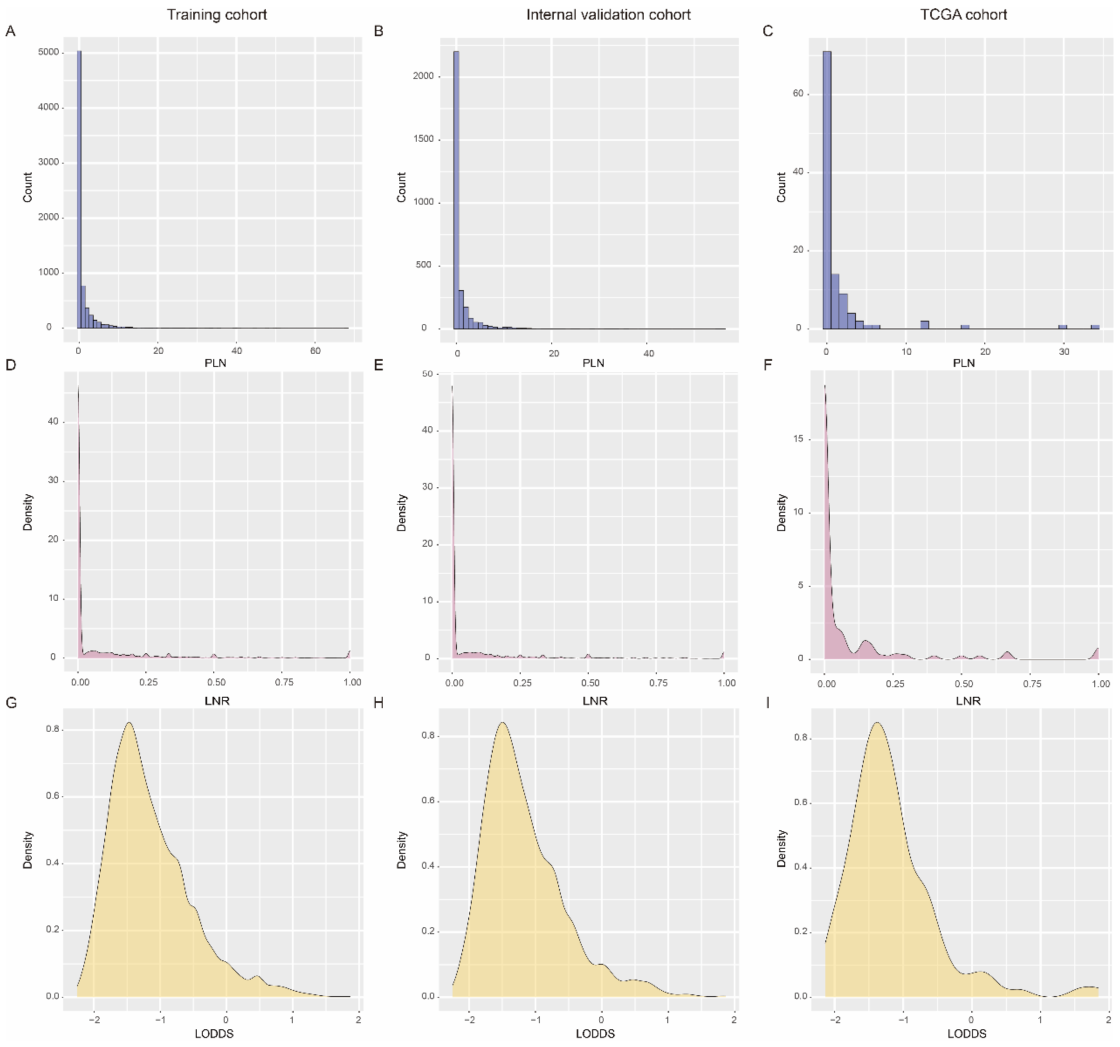

Figure S1. The specific distribution of the lymph node indicators. (A-C) The distribution histograms of PLN in three cohorts. (D-F) The density plots of LNR in three cohorts. (G-I) The density plots of LODDS in three cohorts. TCGA: The Cancer Genome Atlas; PLN: positive lymph node; LNR: lymph node ratio; LODDS: log odds of positive lymph node.
