# Supplementary material for: Prognostic nomogram based on the lymph node metastasis indicators for patients with bladder cancer: A SEER population‐based study and external validation
Source: Cancer Med. 2022 Dec 7;12(6):6853–66. doi: 10.1002/cam4.5475 (PMC10067030; doi:10.1002/cam4.5475)
Supplement: Supplementary file 2 — Figure S2. [file CAM4-12-6853-s003.pdf]

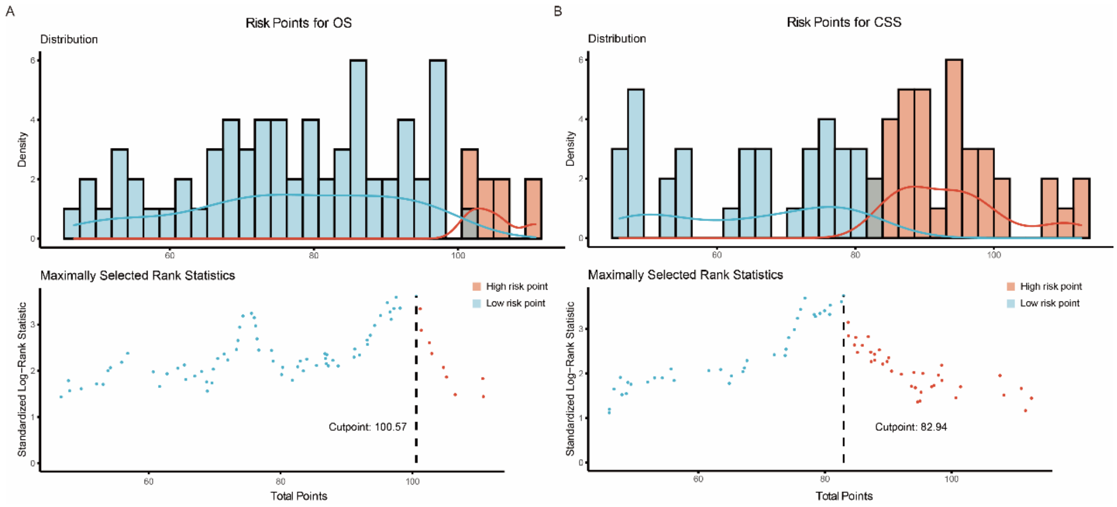

Figure S2. The identification of optimal cutoff of risk points based on the nomogram models for (A) OS and (B) CSS. The scatter plots present the standardized log-rank statistic values for each corresponding expression cutoff. The optimal cutoff with the maximum standard log-rank statistic is marked with a vertical dashed line. The histograms show the density distribution of high- and low- risk groups divided by the optimal cutoff. OS: overall survival; CSS: cause-specific survival.
