# Supplementary material for: Prognostic nomogram based on the lymph node metastasis indicators for patients with bladder cancer: A SEER population‐based study and external validation
Source: Cancer Med. 2022 Dec 7;12(6):6853–66. doi: 10.1002/cam4.5475 (PMC10067030; doi:10.1002/cam4.5475)
Supplement: Supplementary file 3 — Figure S3. [file CAM4-12-6853-s005.pdf]

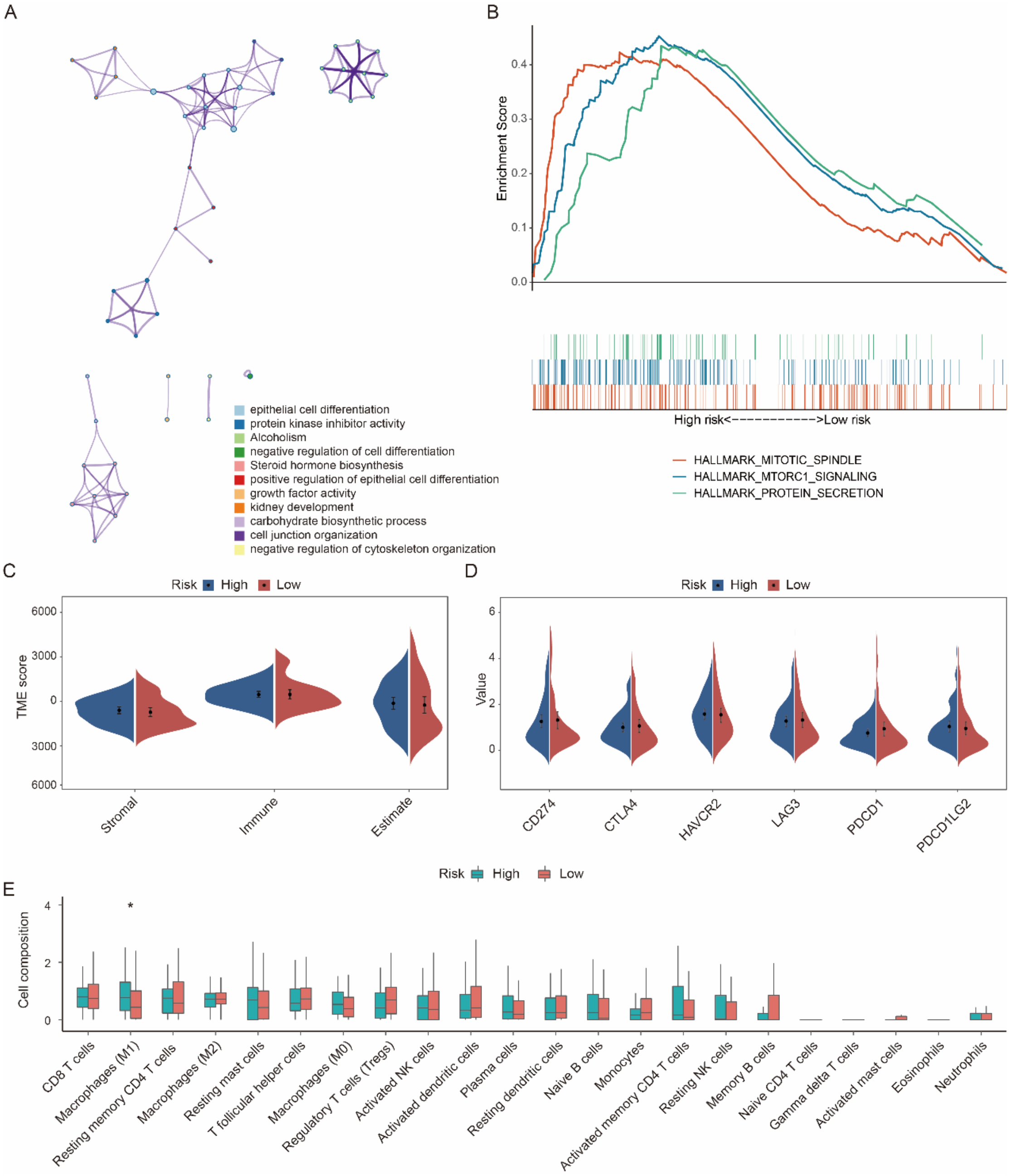

Figure S3. Analyses of pathway enrichment and tumor immunity of the risk classifier stratified by CSS. (A) Network plot of the enriched terms clustered by Metascape. (B) The significantly enriched Hallmark gene sets performed by Gene Set Enrichment Analysis. (C) The stromal and immune scores between two groups. (D) Different expression of ICIs-related genes among risk groups. (E) The difference proportion of tumor-infiltrating cells between two groups. * represented P value < 0.05. CSS: cause-specific survival; ICIs: immune checkpoint inhibitors; TME: tumor microenvironment.
