# Supplementary material for: Prognostic nomogram based on the lymph node metastasis indicators for patients with bladder cancer: A SEER population‐based study and external validation
Source: Cancer Med. 2022 Dec 7;12(6):6853–66. doi: 10.1002/cam4.5475 (PMC10067030; doi:10.1002/cam4.5475)
Supplement: Supplementary file 4 — Table S1. [file CAM4-12-6853-s004.pdf]

Table S1. The detailed AICs of the backward stepwise selection for multivariate regression models.

Endpoint	Filtered Model	Variables	AIC
OS	N classification	Age + Gender + Grade + T + M + N	65514.88
		Age + Grade + T + M + N	65513.18
		Age + T + M + N	65511.93
	PLN	Age + Gender + Grade + T + M + PLN	65767.11
		Age + Gender + T + M + PLN	65765.27
		Age + T + M + PLN	65764.07
	LNR	Age + Gender + Grade + T + M + LNR	65571.17
		Age + Gender + T + M + LNR	65569.18
		Age + T + M + LNR	65567.82
	LODDS	Age + Gender + Grade + T + M + LODDS	65461.96
		Age + Gender + T + M + LODDS	65459.96
		Age + T + M + LODDS	65458.57
CSS	N classification	Gender + Grade + T + M + N	48756.50
		Gender + T + M + N	48755.03
	PLN	Gender + Grade + T + M + PLN	49048.33
	LNR	Gender + Grade + T + M + LNR	48841.40
	LODDS	Gender + Grade + T + M + LODDS	48732.61

Abbreviations: AIC: Akaike Information Criterion; OS: overall survival; CSS: cause-specific survival; PLN: positive lymph node; LNR: lymph node ratio; LODDS: log odds of positive lymph nodes.
