# Supplementary material for: Prognostic nomogram based on the lymph node metastasis indicators for patients with bladder cancer: A SEER population‐based study and external validation
Source: Cancer Med. 2022 Dec 7;12(6):6853–66. doi: 10.1002/cam4.5475 (PMC10067030; doi:10.1002/cam4.5475)
Supplement: Supplementary file 5 — Table S2. [file CAM4-12-6853-s001.pdf]

Table S2. Prognostic efficiency of nomograms for patients with BC in three cohorts

Endpoint	Cohort	C-index	AUC		
			1-year	3-year	5-year
OS	Training	0.705	0.770 (0.756-0.784)	0.768 (0.756-0.779)	0.759 (0.747-0.770)
	Internal validation	0.708	0.753 (0.731-0.775)	0.774 (0.757-0.791)	0.763 (0.745-0.781)
	TCGA	0.633	0.612 (0.477-0.748)	0.723 (0.602-0.844)	0.742 (0.614-0.870)
CSS	Training	0.727	0.783 (0.769-0.798)	0.783 (0.771-0.794)	0.775 (0.763-0.788)
	Internal validation	0.731	0.771 (0.748-0.793)	0.791 (0.774-0.809)	0.783 (0.765-0.801)
	TCGA	0.667	0.693 (0.569-0.817)	0.728 (0.596-0.861)	0.761 (0.608-0.913)

Abbreviations: BC: bladder cancer; C-index: concordance index; AUC: area under the receiver operating characteristic curve; OS: overall survival; CSS: cause-specific survival; TCGA: The Cancer Genome Atlas.
